# Experimental data in support of continuous microwave effect on emulsion polymerization of styrene

**DOI:** 10.1016/j.dib.2015.07.009

**Published:** 2015-07-17

**Authors:** Başak Temur Ergan, Mahmut Bayramoğlu, Seval Özcan

**Affiliations:** Gebze Technical University, Department of Chemical Engineering, Gebze, Kocaeli, Turkey

## Abstract

This article contains original experimental data, figures and methods to the study of Microwave-assisted emulsion polymerization of styrene under the frame of “Enhanced Microwave Synthesis” (EMS), has been examined to investigate the advantages of Microwave (MW) power use in emulsion polymerization (Ergan et al., Eur. Polym. J. 69, 2015, 374–384). For comparative purpose, MW and conventional heating (CH) method experiments were conducted under similar conditions. By externally cooling the reaction vessel with 1,4-dioxane, constant and continuous MW power was successfully applied at isothermal condition during the polymerization. Here we give the MW power calibration data of MW-experimental system, the complete set of the experimental polymerization data and the analysis data obtained from different polymer characterization test devices (GPC, DSC and Viscometer).

**Table:** 

**Specifications Table**	
Subject area	Chemical Engineering
More specific subject area	Microwave reaction systems and Polymer chemistry
Type of data	Table, graph
How data was acquired	Microwave device (Milestone-start-S model), Macrosizer ( Malvern Mastersizer 2000), Gel permeation chromatography (GPC, Agilent 1100 Series), Differential scanning calorimetry (DSC, Perkin Elmer Jade model), Ubbelohde viscometer (capillary diameter, 0.63 mm)
Data format	Calibration data of MW system (raw), polymerization data (raw), Polymer characterization data (analyzed)
Experimental factors	– *Monomer (styrene) was purified by NaOH solution (0.0025M) prior to polymerization* – *Ultrasonic Pre-treatment was applied to prepare an effective emulsion mixture* – *Applied MW power (P*_*nom*_*) was calibrated to evaluate MW power adsorbed in the reaction medium (p) by means of a correction factor* – *Constant and continuous application of MW power was realized at control set point while achieving also isothermal conditions during the polymerization*
Experimental features	– *Emulsion polymerization experiments were conducted by both MW and conventional heating (CH) methods under similar conditions for comparison purpose*
Data source location	Gebze Technical University,Kocaeli,Turkey
Data accessibility	Data are available with this article

**Value of the data**

• The data shows the successful application of continuous and constant MW power during the polymerization while maintaining isothermal condition as well.
• The data provide suitable process conditions for achieving high yields of polystyrene by MW-assisted emulsion polymerization.
• The data provide the proofs for the existence of the “*specific MW effect*”.

## 1 Data, experimental design, materials and methods

Styrene (M) received from Merck was purified with a freshly prepared solution of NaOH (0.0025 M) before using in order to eliminate the inhibitor in styrene. Then, styrene was washed with ultrapure water until the pH was 7. Other chemicals; 1,4-dioxane, Potassium persulfate (KPS), Hydroquinone, Sodium dodecyl sulfate (SDS) were used as received from Merck. Bi-distilled water was used in all the experiments.

According to *one variable at a time* planning technique; temperature (*T*), MW power density (*P*), molar ratios; H_2_O/M, SDS/M, KPS/M and the reaction time (*t*) were investigated as six experimental variables in the ranges 65–85 °C, 0–0.8 kW dm^−3^, 3–9, 0.06–0.1, 0.002–0.005, 7.5–90 min. respectively [1].

**Fig. 1:**
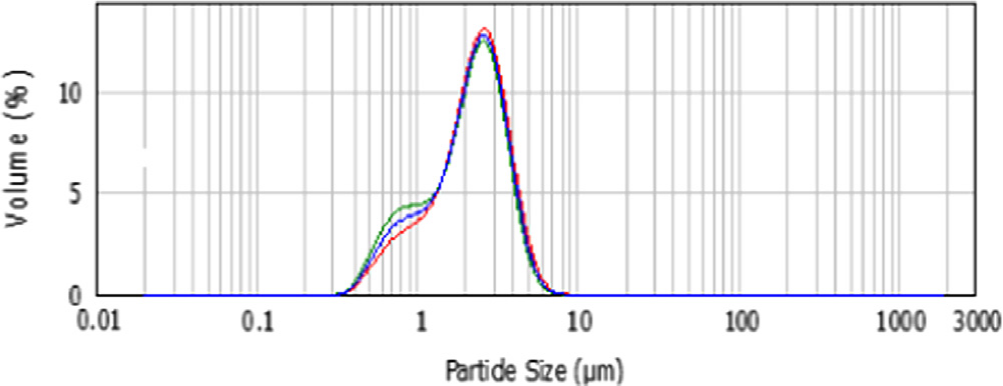
Emulsion particle size distribution.

In a typical run, 60 cm^3^ water, SDS, KPS, and M mixture were load into the jacketed glass reactor and stirred at room temperature for a complete dissolution. Then, ultrasonic pre-treatment was applied to prepare an effective emulsion mixture. Emulsion droplet sizes were measured three times through a Macrosizer device (Malvern Mastersizer 2000, UK) approximately 30 min after the sample preparation. Finally, emulsion droplet size distributions were obtained typically between 0.8 µm and 10 µm as shown in Fig. 1.

**Fig. 2:**
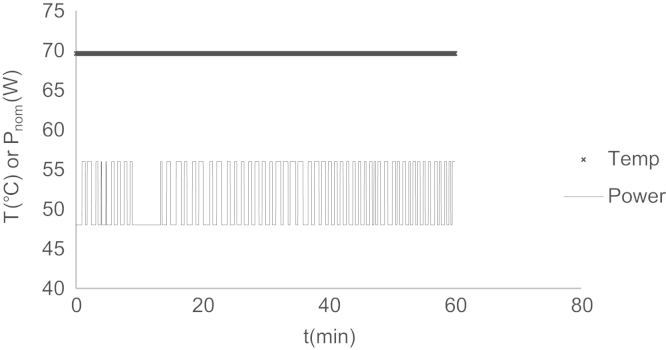
A typical experimental plot (Exp. code: S1-Exp-6).

In this study, a multimode MW reactor (Start-S model, Milestone S.r.l. Sorisole, Italy) was used. During the runs, the Fluoroptic (FO) sensor (accuracy±0.2 °C, ATC-FO-300008 type, Zu electronic, Italy) was dipped in the reactor in a glass capillary sheath. By external circulation of 1,4-dioxane as coolant between jacketed glass reactor and cooling bath, continuous and constant MW energy was applied under isothermal conditions as in our previous studies [2–4]. So, our MW experimental system differs from the literatures which use the cooling system by “air cooling“ [5–8] while applying discontinuous MW power [9–11]. A typical experimental plot with the temperature/MW power data received per 1 s time interval is shown in Fig. 2.

### 1.1 Calibration procedure and data of the microwave power output

According to the IEC 60705 standard method [12–14], empty jacketed glass vessel was weighed, filled with different amount of distilled water and placed into the MW reactor cavity. MW energy (*P*_nom_) was supplied according to amount of water. The water was stirred along the heating period by a magnetic stirrer at 160 rpm. After 60 s, the final temperature of water was measured by Fluoroptic (FO) sensor. Absorbed MW power (*P*) by the vessel, water, magnet and 1,4-dioxane are calculated by means of *Q*=*mc*Δ*T*. The results were given in Table 1. To account for the differences between the absorbed and nominal power values, a correction factor (*p*) is defined as “*P*/*P*_nom_” which is used to calculate the required *P*_nom_ to achieve a given *P* value during the polymerization. Mean value of *p* was calculated as 0.608 under chosen experimental conditions and experimental system (reaction volume: 60 cm^3^) used in this study.

Where *P*: absorbed power (W), *m*: mass of materials (g) (container, water, magnet, 1,4- dioxane), *c*: specific heat capacity of the materials, heating time=60 s, *T*_1_=initial temperature of water (10±0.5 °C), *T*_2_=final temperature of water (approximately ambient temperature).

### 1.2 Experimental data of MW-assisted emulsion polymerization of styrene

Six experimental variables given in Table 2 were investigated and suitable experimental conditions to achieve polymer yield >95% were determined as *T*=75 °C, SDS/M=0.06, KPS/*M*=0.004, H_2_O/*M*=6 and *P*=0.6 kW dm^−3^.

### 1.3 Experimental data of CH-emulsion polymerization of styrene

Five CH experiments were conducted also at the experimental conditions (*T*=75 °C, SDS/*M*=0.06, KPS/*M*=0.004, H_2_O/*M*=6) at different reaction times. The results are presented in Table 3. The comparison of CH and MW experimental results at the same conditions demonstrate the advantage of MW application in term of polymerization time.

### 1.4 Polymer characterization data

MW and CH polymer samples synthesized at the same process conditions were found to have similar structural and thermal characteristics. The analysis data supplied by GPC, DSC and Viscosity instruments is shown in Table 4.

## Figures and Tables

**Table 1:** Calibration data of the microwave power output in the MW system.

Exp. no	*T*1 (°C)	*T*2 (°C)	Δ*T*	Amount of water (g)	*P*_nom_ (W)	*P* (W)	Correction factor (p)
1	9.5	16.2	6.7	40	40	25	0.631
2	9.0	15.5	6.5	50	50	32	0.640
3	9.6	13.5	3.9	30	30	12	0.416
4	9.8	20.9	11.1	30	75	33	0.444
5	10.1	20.3	10.2	50	90	45	0.499
6	9.3	19.7	10.4	40	70	39	0.551
7	9.4	21.5	12.1	80	120	84	0.699
8	9.3	25.0	15.7	50	100	76	0.756
9	9.7	16.9	7.2	60	60	40	0.672
10	9.4	17.7	8.3	80	80	58	0.724
11	9.3	18.3	9.0	90	100	69	0.690
12	9.9	18.7	8.8	100	100	74	0.736
13	9.0	17.8	8.8	100	100	74	0.736
14	9.4	22.7	13.3	100	150	111	0.738
15	9.4	20.0	10.6	60	110	54	0.492
16	9.8	24.8	15.0	60	120	76	0.633
17	9.5	27.1	17.6	70	140	101	0.723
18	9.5	24.3	14.8	70	130	85	0.657
19	9.5	23.6	14.1	110	160	127	0.794
20	9.5	24.5	15.0	150	200	177	0.885
21	9.5	23.7	14.2	200	230	217	0.944
22	9.7	22.6	12.9	250	250	242	0.970
23	9.7	17.8	8.1	60	60	42	0.693
24	9.8	24.9	15.1	60	120	77	0.638
25	9.7	24.8	15.1	60	120	77	0.638
26	9.7	17.9	8.2	60	60	46	0.763
27	9.7	18.0	8.3	60	60	46	0.772
28	9.3	24.7	15.4	150	200	182	0.908
29	9.8	14.2	4.4	60	50	29	0.573
30	9.4	15.5	6.1	60	70	40	0.567
31	9.5	17.6	8.1	60	90	53	0.586
32	9.7	19.5	9.8	60	110	64	0.580
33	9.3	20.5	11.2	60	130	73	0.561
34	9.1	22.6	13.5	60	150	88	0.586
35	9.0	24.6	15.6	60	170	101	0.597
36	9.0	27.8	18.8	60	200	122	0.612
37	9.0	29.3	20.3	60	230	132	0.574
38	9.0	32.0	23.0	60	250	150	0.599

**Table 2:** MW—experimental data.

Exp. code	*t* (min)	*T* (°C)	SDS/M	KPS/M	H_2_O/M	*P* (kW dm^−3^)	Yield %	MW energy (kW h kg^−1^)	Production rate (kg m^−3^ h^−1^)
**S1-exp-4**	**60**	**70.0**	**0.06**	**0.002**	**6**	**0.6**	**92.3**	**7.1**	**131.9**
S1-exp-5	60	70.0	0.08	0.002	6	0.6	91.6	6.5	130.9
S1-exp-6	60	70.0	0.1	0.002	6	0.6	93.2	7.1	133.1
S1-exp-4	60	70.0	0.06	0.002	6	0.6	92.3	7.1	131.9
S3-exp-1	60	70.0	0.06	0.003	6	0.6	93.4	6.8	133.5
**S3-exp-2**	**60**	**70.0**	**0.06**	**0.004**	**6**	**0.6**	**94.3**	**6.5**	**134.7**
S3-exp-3	60	70.0	0.06	0.005	6	0.6	94.0	6.9	134.2
S4-exp-3	60	60.0	0.06	0.004	6	0.6	84.3	7.5	120.4
S4-exp-1	60	65.0	0.06	0.004	6	0.6	93.0	6.9	132.8
S3-exp-2	60	70.0	0.06	0.004	6	0.6	94.3	6.5	134.7
**S4-exp-2**	**60**	**75.0**	**0.06**	**0.004**	**6**	**0.6**	**95.4**	**6.8**	**136.3**
S4-exp-4	60	80.0	0.06	0.004	6	0.6	96.5	6.7	137.8
S4-exp-5	60	85.0	0.06	0.004	6	0.6	96.8	6.4	138.2
S5-exp-13	7.5	75.0	0.06	0.004	6	0.4	70.3	6.3	100.4
S5-exp-9	30	75.0	0.06	0.004	6	0.4	93.6	4.8	133.7
S5-exp-1	65	75.0	0.06	0.004	6	0.4	95.3	5.0	136.2
S5-exp-2	75	75.0	0.06	0.004	6	0.4	95.3	4.6	136.2
S5-exp-3	90	75.0	0.06	0.004	6	0.4	96.6	5.3	137.9
S5-exp-12	7.5	75.0	0.06	0.004	6	0.6	71.3	8.7	101.9
S5-exp-11	15	75.0	0.06	0.004	6	0.6	89.8	7.5	128.3
S5-exp-10	30	75.0	0.06	0.004	6	0.6	93.6	6.5	133.8
S5-exp-4	45	75.0	0.06	0.004	6	0.6	94.9	7.4	135.6
**S4-exp-2**	**60**	**75.0**	**0.06**	**0.004**	**6**	**0.6**	**95.4**	**6.8**	**136.3**
S5-exp-5	75	75.0	0.06	0.004	6	0.6	96.4	6.5	137.7
S5-exp-14	7.5	75.0	0.06	0.004	6	0.8	71.2	13.7	101.7
S5-exp-6	30	75.0	0.06	0.004	6	0.8	93.6	9.6	133.6
S5-exp-7	45	75.0	0.06	0.004	6	0.8	95.6	10.3	136.5
S5-exp-8	60	75.0	0.06	0.004	6	0.8	95.8	8.9	136.8
add-1	7.5	75.0	0.06	0.004	6	0.3	64.4	5.1	91.9
S6-exp-1	90	75.0	0.06	0.004	9	0.4	95.4	7.3	95.4
**S5-exp-3**	**90**	**75.0**	**0.06**	**0.004**	**6**	**0.4**	**96.6**	**5.3**	**137.9**
S6-exp-2	90	75.0	0.06	0.004	3	0.4	97.2	2.9	243.0

The bold style indicate the “suitable experimental conditions” in each serial.

**Table 3:** CH/MW experimental data for comparison purpose.

Method	Exp. code	*t* (min)	*T* (°C)	SDS/M	KPS/M	H_2_O/M	*P* (kW dm^−3^)	Yield %	MW energy (kW h kg^−1^)	Production rate (kg m^−3^ h^−1^)
CH	CH-1	90	75	0.06	0.004	6	0.0	94.6	0.0	135.3
	CH-2	45	75	0.06	0.004	6	0.0	91.2	0.0	130.4
	CH-3	30	75	0.06	0.004	6	0.0	89.7	0.0	128.2
	CH-4	15	75	0.06	0.004	6	0.0	86.4	0.0	123.4
	CH-5	7.5	75	0.06	0.004	6	0.0	51.0	0.0	72.9
MW	add-1	7.5	75	0.06	0.004	6	0.3	64.4	5.1	91.9
	S5-exp-13	7.5	75	0.06	0.004	6	0.4	70.3	6.3	100.4
	S5-exp-12	7.5	75	0.06	0.004	6	0.6	71.3	8.7	101.9
	S5-exp-14	7.5	75	0.06	0.004	6	0.8	71.2	13.7	101.7
	S5-exp-11	15	75	0.06	0.004	6	0.6	89.8	7.5	128.3
	S5-exp-6	30	75	0.06	0.004	6	0.8	93.5	9.6	133.6
	S5-exp-10	30	75	0.06	0.004	6	0.6	93.6	6.5	133.8
	S5-exp-7	45	75	0.06	0.004	6	0.8	95.5	10.3	136.5
	S5-exp-3	90	75	0.06	0.004	6	0.4	96.6	5.3	137.9

**Table 4:** Polymer characterization data of CH and MW experiments at similar process conditions.

Results	CH	MW
*t* (min)	45	90
*T* (°C)	75.0	75.0
SDS/M	0.06	0.06
KPS/M	0.004	0.004
H_2_O/M	6	6
*P* (kW dm^−3^)	–	0.6
*M*_*v*_ (g mol^−1^)	1715,037	1447,770
*M*_*n*_ (g mol^−1^)	1179,000	969,900
*M*_*w*_ (g mol^−1^)	1829,000	1665,700
*M*_*z*_ (g mol^−1^)	2523,300	2447,800
*D*_*p*_	11,336	9,326
*D*	1.5518	1.7174
*T*_*g*_ (°C)	105.6	104.1
*T*_*m*_ (°C)	422	425
*c*_*p*_ (J g^−1 °^C^−1^)	0.365	0.228
